# Loneliness among older Chinese individuals: the status quo and relationships with activity-related factors

**DOI:** 10.1186/s12877-023-04611-9

**Published:** 2024-01-10

**Authors:** Jiazhou Wang, Yueyue Zhou, Qiuxia Zhang, Jing Li, Dehua Zhai, Jia Li, Buxin Han, Zhengkui Liu

**Affiliations:** 1 CAS Key Laboratory of Mental Health, Institute of Psychology, 100101 Beijing, China; 2 Department of Psychology, University of Chinese Academy of Sciences, 100049 Beijing, China; 3 Department of Psychology, Henan University, 475004 Kaifeng, China; 4 China Research Center on Aging, 100011 Beijing, China

**Keywords:** Chinese older population, Loneliness, Activity ability, Activity participation

## Abstract

**Background:**

With the rapid population aging, healthy aging has become a concern for society as a whole. In this study, loneliness and its relationships with activity-related individual factors were examined among older Chinese individuals from the perspective of mental health and daily leisure activities.

**Methods:**

The data were from the fourth investigation of the Sample Survey of the Aged Population in Urban and Rural China, which had a total of 220,506 participants. Activity ability was assessed by the Barthel Activity of Daily Living Index, a self-designed activity type questionnaire was used to evaluate activity participation, and loneliness was measured with a single-item question.

**Results:**

The prevalence of varying degrees of loneliness among Chinese older individuals was 36.6%. The prevalence of loneliness among the older individuals differed significantly by age gender, age, physical health status, annual household income, education level, marital status, living status, ethnic minority status, religious faith and territory of residence. There were differences in activity participation among older Chinese adults in terms of all the demographic factors mentioned above, while there were no significant differences in living status or religious faith, and significant differences in several other demographic factors in terms of activity ability. Self-care ability, as a form of activity ability, and activity participation significantly predicted loneliness among the older participants.

**Conclusion:**

The topic of loneliness among Chinese older individuals is complex and requires greater attention. The buffering effect of activity-related factors on loneliness suggests that old people should improve their activity ability and participate more in daily activities.

## Background

Population aging is becoming an inexorable outcome of population development for the whole world [1]. Meanwhile, aging is a normal and inevitable physiological process for individuals [2]. With retirement and increasing frailty with age, older people experience irreversible changes in their physical and mental health and functional loss, becoming a high-risk group for experiencing loneliness [3]. Loneliness is considered in part a psychological manifestation of social isolation, reflecting an individual’s experience of dissatisfaction with the relationship, frequency, or extent of their social contacts [4]. Research on the emotions of older individuals has found that loneliness is one of the most commonly experienced feelings in old age [5]. Loneliness in older individuals has become a topic of interest that has attracted worldwide attention. In Western society, nearly half of the people in European Union countries believe that loneliness is the main problem faced by the older population [6], multiple studies in the United States have confirmed the universality of loneliness among older individuals [7]. Loneliness among older individuals has become a social fact across borders, cultures and races [8]. As one of the countries with the fastest aging rates and largest older populations in the world, China’s problem of lonely older individuals is particularly prominent [9]. Studies have shown that in recent years, the level and incidence of loneliness among older individuals in China have become increasingly high [10], which is not conducive to the healthy and active aging of older people. Healthy aging is a concept that arose in response to the world’s aging population, which means ensuring the physical, mental and social health and quality of life of elderly people during the aging process [11]. Positive aging is based on healthy aging, with more emphasis on mental health rather than physical health [12]. As an important part of mental health, loneliness is a familiar problem among older individuals alongside population aging, which is important and worth exploring.

Loneliness is a negative emotional state that reflects individual dissatisfaction with one’s own degree of social participation and communication, and it is closely related to mental health [13]. Studies have noted that loneliness often leads to a variety of negative physical and mental consequences; an increase in negative emotional experiences [14] and a decline in cognitive function [15] are both related to loneliness. More seriously, loneliness significantly aggravates morbidity [16] and mortality [17] in older individuals, resulting in an increase in mortality risk that is even comparable to widely recognized risk factors such as lack of exercise and smoking [18]. As an important reference indicator for psychological health, which is one dimension of quality of life [19], preventing loneliness has gradually become a public health priority [20]. Researchers have attempted to reveal the predictive factors of loneliness from various perspectives. In terms of the demographic differences in loneliness among Chinese elderly people, previous studies found risk factors such as disease [21] and widowed status [22], while others found that loneliness was positively correlated with age [23] and negatively correlated with economic level [24]. However, these studies had limited sample sizes, and the results cannot be generalized, either in terms of age or the geographical distribution of participants. An extensive systematic survey that integrated multiple studies on loneliness among older Chinese people determined the main demographic predictors of loneliness in the Chinese older population, namely, gender, age, education level, marital status, economic level, living conditions and health status, and highlighted social support as the key factor in alleviating loneliness [25].

Based on the social characteristics of loneliness, most previous studies have argued that social support can fill the gap between one’s social network environment and one’s demand for social interaction [26], emphasizing the importance of social circumstances connections. However, the cognitive difference model suggests that in reality, while some people do not actually feel lonely despite their frequency or quality of social contact being low, others still feel lonely despite having high extents of social embeddedness [27]. This suggests that considering the factors influencing loneliness solely from the perspective of circumstances is not sufficient. As a subjective feeling, loneliness needs to be described more based on personal experiences.

From the personal perspective, activity theory points out that old people’s participation in daily leisure activities is the basis of and key to inhibiting loneliness, obtaining psychological satisfaction, and promoting healthy longevity [28]. Based on this theory, participating in various daily leisure activities has a positive effect on reducing loneliness in older individuals [29]. Daily leisure activities refer to meaningful activities in daily life that are not aimed at making a living or making a profit, but are of personal interest. From the perspective of the participants number, it can be divided into group activities and solitary self-entertainment activities. Older people who participate in more activities have better activity abilities [30], lower morbidity and mortality [31]. This is because as individuals grow older, they may lose their original social role and relevant sense of purpose and identity due to reduced opportunities for social contact owing to retirement and frailty [32]. Activity participation can help people adapt to these changes by promoting a new identity and socialization, providing a sense of continuity, belonging and connection that enables people to integrate into their new life [33]. What’s more, the feature of leisure can help individuals relax both physically and mentally, so as to promote the physical and mental health and cultural development of the old people more comprehensively [34].

More importantly, a review of Western studies on interventions for loneliness pointed out that, contrary to most of the previous views, not only can group activities (activities that require multiple participants) alleviate loneliness, but solitary self-entertainment activities (activities that can be completed by oneself) also have a protective effect against loneliness [35]. This finding coincides with previous results regarding the dual attributes of activity participation among the Chinese older population, namely, “group communication” and “self-recreation” [36]. From this research perspective on activity, activity participation is divided into two types, i.e., group and individual, which correspond to group communication and self-recreation, respectively. Group communication highlights the characteristics of social interaction and extroversion, such as playing chess/card games and going to the cinema/theatre, while self-recreation mainly highlights the characteristics of egoism and introversion, such as reading and watching TV or listening to the radio alone at home. In self-recreation, the peacefulness, solitude and lack of interchanges does not equate to social isolation but rather to an internalized, self-paced and socially continuous form of social participation [37]. This indicates the dual nature of the influencing mechanism of loneliness; that is, loneliness is affected not only by the strength of interpersonal relationships but also by a strong self-feeling [38]. Overall, activity participation is significant for older people’s mental health and longevity. Previous studies on Chinese older people’s activity participation have mostly focused on the activity content [39] and participation purpose [40], however, demographic differences in activity participation have not been clarified and require further exploration. Although previous studies have mentioned the relationship between occupation [41] and economic level [42] with activity participation, the depth and breadth of their findings need to be further expanded.

While it is generally believed that activity participation lessens loneliness, previous studies on individual activity ability and loneliness have yielded inconsistent results. Some studies have shown that poorer activity abilities are associated with higher loneliness scores [43]. However, other studies have found the opposite, showing that poor mobility may also relate to lower loneliness because of the greater social contact provided by external nursing and assistance [44]. In summary, previous studies have revealed inconsistent results on the relationship between activities and loneliness, and most of them have reported only correlations. In addition, existing studies on the demographic differences in activity ability still remain at the simple level of individual diversity (gender, age, etc.) [3] and health status [23], and further exploration is needed to determine the actual activity ability of different types of older people. The predictive effect of activity-related factors on loneliness after controlling demographic factors is still unclear. Therefore, this study proposes another perspective of loneliness, that is, that loneliness in older individuals should not only be concerned with the perspective of social relations but also be understood from the aspect of the individual. Aiming to make an indigenization interpretation of predictors among older people’s loneliness from the perspective of activity in the Chinese cultural context, we simultaneously explore healthy aging and positive aging in China and contribute to efforts to address global aging.

This study uses an epidemiological method to examine the status and their demographic difference of loneliness and activity-related factors in older Chinese individuals and focuses on the relationship between loneliness and activity-related factors. Activity-related factors are divided into subjective factors and objective factors. Subjective factors reflect the willingness and enthusiasm of older people to participate in activities based on the type and number of activities, while activity ability, including self-care ability and ambulation ability, represents objective factors. Evaluation of the physical activity level is used to determine whether participation in activities can be carried out effectively. The purpose of this study was to understand the activity participation, activity ability and loneliness of older Chinese individuals, as well as the relationship between daily leisure activities and loneliness, to provide a scientific basis for subsequent studies. Our research questions were as follows. (1) What are the status quo of loneliness, activity ability, activity participation and demographic differences among older Chinese people? (2) What is the relationship between activity-related factors and loneliness among older people in China? Based on existing theories and previous research, our hypothesis was as follows. (1) The level of loneliness, activity ability and activity participation differed significantly across various demographic variables. (2) The better the activity ability of older people is, the more activities they participate in, and the lower their level of loneliness.

## Methods

### Participants

The data of this study were sourced from the fourth investigation of the Sample Survey of the Aged Population in Urban and Rural China (SSAPUR). This survey is a large-scale and important thematic national survey jointly carried out by the Chinese National Committee on Aging, the Ministry of Civil Affairs, and the Ministry of Finance. It aims to understand the basic living conditions of urban and rural older people in China and provide scientific support for the country and governments at all levels to coordinate and formulate strategies, plans, and policies to address population aging. In China, the SSAPUR is the largest database on older people. The data have high scientific credibility and representativeness and can be used to represent the overall situation of older individuals in China. The main respondents are older individuals aged 60 and above in Chinese mainland who were selected during the survey period, and they are distributed in all provincial administrative regions of China except Hong Kong, Macao and Taiwan. A total of 220,506 valid questionnaires were collected in the study. The gender ratio of the study sample is 1:1.09, and the average age of the participants is 70.08 years (SD = 7.85, range = 60–110 years).

### Procedures

This cross-sectional study was conducted in China from August 1 to 31, 2015, using a stratified, multistage probability proportional to size sampling method. The sampling approach for the entire data collection process was as follows. The total sample size was calculated using the proportional estimation method, and then a sampling ratio of 1/1000 was determined based on the total number of older people in China. The sample size was allocated according to the proportion of the elderly population in each province to the Chinese population. The sampling amount of counties, townships, and communities was determined, and 462 counties were selected. Based on the multistage probability proportional to size sampling method, 4 communities from each township and 4 townships from each county were selected; thirty participants were selected from each community. The selected neighborhood/village committee assigned standardized trained survey assistants to conduct a questionnaire survey with the selected older people. The questionnaire survey was conducted in the participants’ home or community activity room. The survey assistant and the participants were face-to-face during the whole survey process, accompanied by the primary caregiver, the survey assistant read the questions, and the participants answered the questions. Participants were informed that their responses would be kept completely confidential. Participation in the survey was completely voluntary. Before the implementation of the survey, all the participants and their primary caregiver signed an informed consent form, and questionnaires were retrieved immediately after the survey. All study procedures were approved by the National Bureau of Statistics of China (approval number: [2014]No. 87).

### Measures

Loneliness. The question to assess loneliness among older adults in the survey was “Do you feel lonely?” Through a self-assessment method, the participants’ loneliness level was divided into three levels based on three answer options to the above question: frequently, sometimes and never. In the subsequent analysis, responses of often and sometimes were merged into the loneliness group, and responses of never were defined as the non-loneliness group, thus forming a dichotomous loneliness variable.

Activity ability. The Barthel Activity of Daily Living (ADL) Index was used to assess the objective activity ability of the older participants [45]. This index is a 10-item scale consisting of two parts, the first part assesses self-care ability and the second part assesses ambulation ability. The items are rated on a 3-point Likert scale, with scores of 0, 5, and 10 representing unable, with help and independent, respectively. The total score ranges from 0 to 100, with higher scores indicating better mobility. In this study, based on the difference between older individuals and clinical patients, the items for grooming and climbing stairs were deleted, resulting in a total score of 80 points for this study. The Cronbach’s α coefficient of the scale was 0.89.

Activity participation. The subjective willingness of the older individuals to participate in activities was evaluated by the type and number of daily leisure activities participated in. The SSAPUR questionnaire listed a total of 11 kinds of daily leisure activities, which comprehensively covered the main daily leisure activities of older Chinese individuals, and asked the respondents to indicate whether they often participated in the activities [46]. Based on previous research [36], this variable was specifically divided into two categories according to the presence or absence of social interaction: group communication and self-recreation. Among them, group communication includes going to the cinema/theatre, dancing, playing ball game, playing chess/card games and keeping pets; Self-recreation includes watching TV/listening to the radio, reading, jogging, doing fitness exercises, painting/calligraphy. Each item was assigned a score of 1, the total score of group interaction activities was 0–5, and the total score of self-recreation activities was 0–6. A higher score reflected a greater number of activities participated in and higher enthusiasm for activity.

Demographic variables. Considering the possible demographic differences among the older participants and the recognized influencing factors for loneliness in the older population in previous studies, we also measured several important demographic variables, such as gender, age, education level, marital status, annual household income level, living status, region of residence (east-central-west), area of residence (urban-rural), ethnicity, religious faith and physical health. The specific variable descriptions are shown in Table 1.

### Analysis plan

All statistical analyses were performed using IBM SPSS 25.0 statistics (IBM, Armonk, NY, USA). First, the participants were divided into loneliness and non-loneliness, according to the above criteria. Second, descriptive statistics were calculated for the demographic characteristics, and the chi-square test was used to analyze the differences in the proportion of older individuals with different levels of loneliness by different demographic variables. A t test was used to test whether loneliness was different at multiple levels of demographic continuous variables. Then, t tests and one-way analysis of variance were used to test whether activity ability and activity participation were different at multiple levels of demographic variables. Finally, to determine whether activity participation and ablility were protective factors against loneliness, we performed binary logistic regression on the whole sample with activity participation and ability as independent variables and loneliness as the dependent variable after controlling for relevant demographic variables.

## Results

### Basic information regarding the demographic characteristics of the older Chinese individuals’ activity ability, activity participation and loneliness

**Table 1:** Differences in loneliness by demographic variables

Characteristic	Overall cohort (n = 220,506)	Non-loneliness (n = 139,719)	Loneliness (n = 80,787)	*X* ^*2*^
	Number (%)	Number (%)	Number (%)	
Gender				1157.446^***^
Male	105,371(47.8)	31,826(50.5)	34,760(43.0)	
Female	115,135(52.2)	33,641(49.5)	46,027(57.0)	
Educational level				
Illiteracy	64,944(29.5)	33,118(23.8)	31,826(39.5)	9392.038^***^
Primary school	90,861(41.3)	57,220(41.1)	33,641(41.8)	
Junior high school	41,487(18.9)	30,790(22.1)	10,697(13.3)	
High/polytechnic school	15,616(7.1)	12,387(8.9)	3229(4.0)	
Junior college	4496(2.0)	3778(2.7)	718(0.9)	
University or above	2401(1.1)	2027(1.5)	374(0.5)	
Ethnic minority				615.388^***^
Yes	14,111(6.4)	7569(5.4)	6542(8.1)	
No	206,164(93.6)	132,025(94.6)	74,139(91.9)	
Region of residence				1273.023^***^
East	99,258(45.0)	66,905(47.9)	32,353(40.0)	
Central	71,150(32.3)	42,599(30.5)	28,551(35.3)	
West	50,098(22.7)	30,215(21.6)	19,883(24.6)	
Area of residence				4611.290^***^
Urban	114,911(52.1)	80,486(57.6)	34,425(42.6)	
Rural	105,595(47.9)	59,233(42.2)	46,362(57.4)	
Living status				17166.499^***^
Living alone	33,091(15.9)	10,692(8.0)	22,399(29.9)	
Living with others	174,716(84.1)	122,324(92.0)	52,392(70.1)	
Religious faith				127.986^***^
Yes	40,591(18.6)	24,739(17.9)	15,852(19.8)	
No	177,919(81.4)	113,770(82.1)	64,149(80.2)	
Marital status				30478.901^***^
Married/Remarried	152,223(71.8)	113,745(84.8)	38,478(49.4)	
Widowed/Divorced/Single	59,910(28.2)	20,447(15.2)	39,463(50.6)	

Note. *** *p* < 0.001; X^2^ = chi-square; The percentage in Table 1 is an effective percentage, and the total number varies slightly among the variables because some variables have a few missing values

The results showed that 36.6% of the older participants had some degree of loneliness, with 30.3% sometimes feeling lonely and 6.3% often feeling lonely. The demographic characteristics of the older participants who did and did not report loneliness were as follows: Compared with the non-loneliness group, the loneliness group was significantly older (mean ± SD: 71.94 ± 8.35 vs. 69.00 ± 7.34, t = 83.073, p < 0.001), had significantly lower annual household income (mean ± SD: 25.69 ± 30.18 vs. 43.17 ± 44.39, t = -108.585, p < 0.001) and physical health status (mean ± SD: 5.04 ± 1.66 vs. 6.14 ± 1.72, t = 145.913, p < 0.001). Table 1 presents in terms of detail the differences of loneliness in various demographic variables. There were significantly different ratios between the participants who did and did not report loneliness by all demographic categorical characteristics. Elaborate results are shown in Table 1.

**Table 2:** Demographic characteristics of activity participation and activity ability

Characteristic	Activity Participation	*t/F*	Activity Ability	*t/F*
	M ± SD		M ± SD	
Gender		51.820^***^		15.782^***^
Male	2.20 ± 1.43		77.12 ± 10.09	
Female	1.88 ± 1.36		76.38 ± 11.28	
Educational level		12823.891^***^		478.226^***^
Illiteracy	1.34 ± 0.96		75.07 ± 12.96	
Primary school	1.88 ± 1.18		77.08 ± 10.04	
Junior high school	2.68 ± 1.47		77.93 ± 8.68	
High/polytechnic school	3.29 ± 1.58		77.94 ± 8.96	
Junior college	3.93 ± 1.56		78.31 ± 8.53	
University or above	3.94 ± 1.52		78.21 ± 8.37	
Ethnic minority		-31.181^***^		-5.205^***^
Yes	1.69 ± 1.32		76.25 ± 10.94	
No	2.06 ± 1.40		76.77 ± 10.72	
Region of residence		471.338^***^		253.658^***^
East	2.13 ± 1.40		77.32 ± 10.09	
Central	1.92 ± 1.32		76.26 ± 11.45	
West	2.00 ± 1.48		76.24 ± 10.88	
Area of residence		169.273^***^		13.280^***^
Urban	2.49 ± 1.52		77.03 ± 10.59	
Rural	1.54 ± 1.05		76.41 ± 10.88	
Living status		54.170^***^		0.346
Living alone	1.70 ± 1.26		76.81 ± 9.66	
Living with others	2.12 ± 1.42		76.83 ± 10.72	
Religious faith		-19.391^***^		1.349
Yes	1.92 ± 1.34		76.8 ± 10.33	
No	2.06 ± 1.41		76.72 ± 10.83	
Marital status		83.907^***^		42.247^***^
Married/Remarried	2.18 ± 1.43		77.46 ± 9.45	
Widowed/Divorced/Single	1.65 ± 1.25		74.85 ± 13.35	

Note. *** *p* < 0.001; M ± SD = Mean ± Standard Deviation; *t* is the statistical value of student’s t test; *F* is the statistical value of F test

There were significant differences in the two types of activity participation among older Chinese individuals. Only 22.9% of the older participants participated in group communication activities, while more than 91.6% of the older participants participated in self-recreation activities (X^2^ = 3435.474, P < 0.001). Among the group communication activities, the activity with the highest participation rate was playing chess/card games (13.5%). Regarding self-recreation activities, the activity with the highest participation rate was watching TV/listening to the radio (89.0%). Activity ability showed a significant positive association with annual household income (r = 0.044, P < 0.001) and negative associations with age (r=-0.235, P < 0.001) and physical health status (r=-0.446, P < 0.001). Regarding activity ability, there was a significant difference between the self-care ability and ambulation ability of the older Chinese individuals: 15.2% of the older participants had one or more impaired self-care abilities, while only 6.2% of the older participants had one or more impaired ambulation abilities (X^2^ = 66159.729, P < 0.001). The self-care ability with the highest rate of impairment was bathing (9.7%), and the ambulation ability with the highest rate of impairment was walking (5.4%). Activity participation was significantly positively associated with annual household income (r = 0.353, P < 0.001) and physical health status (r=-0.324, P < 0.001), and negatively associated with age (r=-0.151, P < 0.001). The activity ability and activity participation scores of the older Chinese individuals at the levels of different demographic variables and additional specific information about this sample are shown in Table 2.

### Effects of activity ability and activity participation on loneliness

To further explore the relationship between loneliness and activity-related factors in older Chinese individuals, after controlling for demographic variables, a binary logistic regression analysis was performed for the whole sample. See Table 3 for other details.

**Table 3:** Predictors of loneliness

Characteristic	B	SE	Wald	OR	95% CI
Activity ability					
Self-care ability	-0.006	0.001	25.212	0.994^***^	0.992,0.996
Ambulation ability	-0.001	0.003	0.058	0.999	0.993,1.005
Activity participation					
Group communication	-0.133	0.012	124.666	0.875^***^	0.855,0.896
Self-recreation	-0.128	0.006	387.822	0.880^***^	0.869,0.891

Note. *** *p* < 0.001. OR = odds ratio; CI = confidence interval

## Discussion

This study was a national survey on loneliness and activity-related individual factors among Chinese older individuals. Compared with the older individuals who did not report loneliness, those who did report loneliness were older, had lower income and worse physical health status, and were more likely to be female, illiterate, an ethnic minority, living in central and rural areas, living alone, religious, and not married. There were significant differences in activity participation among older individuals by gender, age, marital status, living status, physical health level, income level, education level, region/area of residence, ethnicity and religious faith. Activity ability varied significantly by gender, age, marital status, physical health level, income level, education level, region/area of residence and ethnicity. Low activity participation and low self-care ability, as a type of activity ability, were risk factors for loneliness.

### Loneliness among older Chinese individuals

The results of this study show that 36.6% of the older participants experienced varying degrees of loneliness, which was similar to previous Chinese studies. Due to different reasons, such as the survey completion period, cultural context and research methods, the findings on the prevalence of loneliness among older individuals differ substantially. The prevalence of loneliness among Chinese older individuals is approximately 30% (35.3%, [47]; 28.0%, [48]; 25.0%, [49] ), which is lower than the results of Western studies (35.3%, [50]; 28.0%, [23]; 25.0%, [51] ). This result may be due to two reasons. First, the interpretation of loneliness needs to be based on cultural background. In the past few decades, China’s “Tide of Rural Workers” and the implementation of the one-child policy have produced many empty nesters in the older population [52]. Their adaptation to living alone for years and the idea of mutual help in the Eastern collectivist culture have eased the loneliness of Chinese older people. Moreover, the reason for the lower self-reported loneliness may be that older adults are less willing to self-disclose, because the word loneliness often has a negative connotation, and older individuals may be ashamed to admit their loneliness because of social pressure and exclusion [53].

In this study, there were significant differences in the distribution proportion of lonely and non-lonely old people in each demographic variable. There was a significant gender difference in loneliness among the older participants, with more women than men reporting loneliness, a finding that has been relatively stable in previous studies and due to multiple reasons. Genetically and hormonally, women are biologically more sensitive to negative emotions such as loneliness [54]. Moreover, women are more able to express their lonely feelings openly than men because society has a higher tolerance and acceptance of negative emotional expression for women [55]. Finally, socially speaking, women’s generally longer life expectancy puts them at greater risk of becoming widowed and experiencing other losses [56]. Older individuals who report loneliness tend to be older than those who do not. The underlying cause of loneliness is the reduced activity and social connection caused by the physical and mental dysfunction associated with aging [57] as well as the loss of one’s social role [58] and the increased possibility of bereavement [59]. The non-loneliness group in this study had better physical health status than the loneliness group. When older people’s viability is impaired, their social communication and interpersonal communication are passively reduced, naturally resulting in a greater probability of feeling loneliness [57]. In addition, not only the objective physical health status is closely related to loneliness, but also the subjective perceived poor physical health status and insufficient physical function will also increase loneliness [60]. Consistent with expectations, those with higher education levels and income showed lower levels of loneliness. Specifically, individuals with higher income have less life stress and less financial conflict and thus have more time and energy to focus on and improve their emotional and psychological condition [61]. While individuals with lower income have fewer economic resources, the frequency and level of their daily leisure activities will be limited, and fewer social resources will be used to alleviate loneliness [62]. Regarding education level, lonely older individuals were more concentrated in the middle and lower levels because compared with those with high education levels, those with low education levels have a poor ability to make social connections [25], while those with higher education levels have better awareness and ability to recognize and ameliorate their negative emotions. Compared with lonely older individuals, nonlonely older individuals were more likely to be married, which fully demonstrates that marriage is a protective factor against loneliness. The spouse is a powerful source of social support [63]. Accordingly, the loss of spouse is a serious negative life event for the old people. Without a spouse to confide in and chat with, older individuals are more likely to experience emptiness and helplessness [64]. The proportion of lonely older individuals living alone was higher than that of nonlonely older individuals. This is because older people living alone lack the company and care of their families, and they are more likely to feel lonely in the face of aging and loss [65]. For the vast majority of people, the family is the safest comfortable environment for individuals, and the respect and love derived from the family can give them the gratification of resisting loneliness [66]. In terms of the region and area of residence, compared to lonely older individuals, the proportion of nonlonely older individuals in socially and economically developed regions was higher, which is related to regional development differences. In urban areas and the more developed eastern regions, policies targeting older people’s mental health care are more comprehensive, and older people’s spiritual and cultural lives are also more diverse [67]. More diverse retirement activities, more convenient medical conditions and infrastructure are all very effective in preventing loneliness [68]. In addition, there were more ethnic minorities and religious people among the lonely older participants in this study, which is also related to the territorial characteristics where they live. Ethnic minorities usually live in relatively remote rural areas. Meanwhile, people with religious beliefs are more distributed among ethnic minorities. A more monotonous life was associated with higher feelings of loneliness.

### Demographic differences in activity-related factors

Activity participation scores were significantly different across all demographic variables. More than 90% of the older Chinese individuals in this study participated in at least one activity, with men being significantly more involved than women. This finding may be due to the fact that women interact more with their families in daily life, while men prefer to interact with friends who have similar interests and characteristics with them [69]. Older people who were younger and healthier were more likely to participate in more activities. Because younger individual has better objective physical conditions to support participation in activities [70]. Socioeconomic status is closely related to activity participation, and the two most commonly used indicators of socioeconomic status are education and income [58]. Specifically, more highly educated people have more powerful social support than people with lower levels of education [71], and income directly affects the frequency of participation in and grade of paid activities [72]. Moreover, the differences in economic development between different regions may be the reason behind the significant difference in the activity participation of the older participants living in different regions. Unexpectedly, older individuals who were single, divorced, widowed or living alone did not participate in more activities to compensate for the lack of spouse or cohabitant emotional support. A possible explanation is that the spiritual emptiness of older individuals may make it difficult to engage in normal social life [73], so there is a lack of internal drive and external support for the willingness to participate in activities. The reason why religious and ethnic minority older people had lower activity participation scores is that they were more distributed in rural areas and regions with lower economic levels, the rhythm and content of their daily lives are more fixed and monotonous because of the limitation by minority customs, so their activity participation is lower [74].

Activity ability scores differed significantly across all demographic variables except living status and religious faith, the possible reasons are as follows. The poor activity ability of minority older individuals in this study may have been caused by the underdeveloped medical infrastructure and inconvenient travel conditions in remote areas. In response, older individuals in the underdeveloped central and west regions and rural areas also had worse activity ability. Unlike the lower activity participation scores of religious old people, there was no significant difference in activity ability score among those with or without religious faith. This may be because, compared with daily activities, religious old people are more involved in religious activities (such as chant scriptures, thurification and zazen, etc.), while the religious activities for old people are usually more moderate and do not require much physical strength [75].The economic conditions of older individuals are also positively correlated with their activity ability [76]. Good economic conditions provide a material basis for the maintenance of good activity ability. Education level is also related to better activity ability. This result may be explained by the fact that well-educated older individuals also have a stronger awareness of attention to their bodies [77]. Single, divorced or widowed older people have less activity ability than married older people, which may be because spouses stand together in times of adversity and can provide physical help and emotional support for individuals to maintain or even restore their mobility. There was no significant correlation between older individuals’ living status and their activity ability. Older individuals who live alone may be more concerned about their physical health condition due to the lack of attention from cohabitants. Only good activity ability can ensure their life independence without company, and this concern can quickly compensate for the decline in activity ability level [78]. In line with previous studies [79], the significant decrease in activity ability with age. Individual aging is inevitably accompanied by the psysical degeneration [80, 81]. When an individual has health problems, its activity ability will naturally decline. Women had lower activity ability levels than men in this study, probably due to more frequent chronic diseases caused by female fertility and menopause [82].

### Predictive roles of activity ability and activity participation on loneliness

Self-care ability, as a type of activity ability, significantly predicted loneliness in older individuals, and there are two possible influencing pathways. First, activity ability contributes to the establishment and maintenance of social connections in daily life [83], which is necessary for older individuals to seek communication and contact. Basic self-care ability can help older individuals maintain a relatively good spiritual outlook, which is a prerequisite for individuals to integrate into society [84]. Second, a lack of self-care ability may reduce the capacity for reciprocity in interactions, which is not conducive to the sustenance of interindividual good relationships and can even lead to tension and decreased relationship quality [85]. However, why does ambulation ability not significantly predict loneliness in older individuals? This may be because, compared with the loss of self-care ability, which can be compensated by old people self, the loss of ambulation ability requires more external help, and such external help provides more social contact [44]. The external support that older people receive thus mitigates perceived loneliness to some extent.

Activity participation is a protective factor for loneliness, and increasing activity participation can alleviate loneliness. First, activities can be used to resist loneliness. Activities occupy individual time and energy, so that individuals ruminate less about their loneliness [86]. Second, activity may function as social zeitgebers, prompting older people’s circadian rhythms to become more regular, thereby stabilizing mood and relieving loneliness [87]. Specifically, group communication activities encourage a healthy positive mindset by providing a sense of belonging and connection [88]. Previous research have found that even non-exercise gentle group activities have benefits for individual health and survival [89]. While self-recreation activities can not only divert the old people’s attention from loneliness [90], but also alleviate loneliness by increasing the sense of control over life and inducing positive self-cognition [91].

### Advantages and limitations

The main improvements in this study are as follows. First, our study settles the debate on the inconsistent relationship between loneliness and activity-related factors in different previous studies by confirming a positive correlation of activity ability and participation with loneliness. Second, among Chinese studies, our sample size is unprecedented, which makes our findings more representative and persuasive. Finally, compared with previous study results that only briefly reported the correlation between activity-related factors and loneliness, this study proposes a new perspective, which is to explore activity-related factors from an individual perspective. Activity-related factors were divided into a subjective dimension, activity participation, and an objective dimension, activity ability, and further refined into 4 specific factors. On this basis, the relationship between activity-related factors and loneliness was discussed in depth, which further enriches the research content.

However, the present study also has several limitations. First, this study had a cross-sectional design, so it could not establish a causal relationship between activity and loneliness outcomes. The use of a longitudinal design in future research may be more conducive to the evaluation of causality. Second, a single-item loneliness measurement may be too ambiguous to deeply discuss the connotations of different dimensions of loneliness, although previous research has shown that single-item measurements are closely related to more complex scales [92]. Finally, all variables were measured using a self-assessment questionnaire, which is prone to report bias, it is still necessary to use structured clinical interviews for the subsequent researches.

### Practical implications

Previous studies lack details on the loneliness status and activity-related environmental factors of older Chinese individuals. As one of the countries with the fastest aging rates, information on older Chinese individuals’ activity ability, activity participation and loneliness can provide practical and useful references for other countries’ response measures to aging. Surveying demographic differences to identify high-risk population characteristics can provide the basis for the promulgation of countermeasures. At the micro level, the study suggests that we should increase attention to disadvantaged groups such as older women. At the macro level, the study also helps better understand the daily condition and psychological status of older Chinese individuals and provide theoretical evidence for the prevention, treatment, and intervention of loneliness in older individuals. The successful prediction of the effects of self-care ability and activity participation on loneliness suggests that in the future, public health policies need to pay more attention to the design and construction of public activity venues and facilities to encourage older people to participate more in exercise and activities. It is very important for adhering to the positive concept of aging and promoting healthy aging.

## Conclusion

In China, compared with older individuals who did not report loneliness, those who did report loneliness were more likely to be female, illiterate, unmarried, an ethnic minority, living in central and rural areas, living alone, and older, have a lower income, and have worse physical health status. Moreover, the activity ability and activity participation of different types of older people were also very different. In general, older people who are more actively involved in activities and have better self-care abilities are more likely not to be lonely. Everyone grows old, ocusing on the older population means focusing on everyone’s future. Although the situation of loneliness among older people in China may not tend to worsen, the negative effects of loneliness on high-risk groups cannot be ignored, which makes concern and intervention for high-risk groups more urgent and requires people to pay more attention. Surveys on the activity ability and activity participation of elderly people reveal great differences in the content and quality of life between urban and rural areas, between east and west, and between people of different economic levels. Similarly, this helps to remind the relevant units to focus on groups with poor activity ability and provide more help to groups with low activity participation. Finally, the protective effect of activity-related factors on loneliness suggests that we can reduce the risk of loneliness by propagating the importance of activities and encouraging older people to be active through valuing activity abilities and participating more in activities to further improve their quality of life and strive to achieve healthy and active aging.

## Data Availability

The datasets used during the current study are available from the corresponding author on reasonable request.

## Declarations

Approval number: [2014]No. 87.
