# Correction to: Loneliness among older Chinese individuals: the status quo and relationships with activity-related factors

**DOI:** 10.1186/s12877-024-04821-9

**Published:** 2024-03-19

**Authors:** Jiazhou Wang, Yueyue Zhou, Qiuxia Zhang, Jing Li, Dehua Zhai, Jia Li, Buxin Han, Zhengkui Liu

**Affiliations:** 1 CAS Key Laboratory of Mental Health, Institute of Psychology, 100101 Beijing, China; 2 Department of Psychology, University of Chinese Academy of Sciences, 100049 Beijing, China; 3 Department of Psychology, Henan University, 475004 Kaifeng, China; 4 China Research Center on Aging, 100011 Beijing, China

After publication of this article, the authors reported that Jiazhou Wang was incorrectly denoted as corresponding author.

The original article has been corrected. 

